# The "Star" on Vivisection

**Published:** 1898-02-19

**Authors:** 


### THE "STAR" ON VIVISECTION.

We have no desire to enter into any controversy with the *Star* in regard to the question of experiments upon animals. The *Star* appears to have got its brief, and no doubt will go on reasserting its old ideas, whatever anyone says. Still it may be worth while to point out that when it states that we defend vivisection "on the ground that there is 'on every hand a perfect ocean of cruelty,' and that therefore the vivisector is no worse than" &c., it entirely misrepresents our position. Nor do we think the *Star* is quite fair to its readers in the "facts" it gives for their edification.

It is all very well to tell ignorant people that 5,297 out of a total of 7,500 experiments performed in 1896 were done without anæsthetics. It would, however, have shown a greater desire to tell its readers the whole truth if the *Star* had added that all of these 5,297 experiments, mentioned as having been performed without anæsthetics, were of the nature of inoculations, hypodermic injections, and similar proceedings, in regard to which the inspector under the Act says: "It would be cruel rather than otherwise to anæsthetise an animal before subjecting it to the trivial operation of a prick with a needle."

Then we think it would not have been amiss if the *Star*, in addition to telling its readers of the "alarming increase" in the number of licences and experiments, had also told them the reason of this increase. The reasons for which these inoculations are done is given in a Parliamentary return as, the study of the organisms by which disease is produced; the diagnosis of disease in man and animals; the determination of the necessity or otherwise of surgical interference, and to decide whether valuable herds of animals shall be sacrificed or preserved; and, finally, the testing of anti-toxins previous to their employment upon men. Of course, the *Star* may ask us to throw away anti-toxins and all our modern knowledge as to the treatment of disease. But we, on the other hand, decline to do so. There are hundreds of children alive in London at the present time whose lives, so far as the evidence goes, have been saved by the use of diphtheria anti-toxin, a drug which can only be given in a haphazard, blindfold fashion, unless it is previously tested upon animals. Our position in regard to vivisection hinges upon this, that our main work is the relief of human suffering, and that experiments on animals increase our power of giving this relief. If the community prefers to suffer that is its own look out. In the meantime we think a little common sense might be of service, and perhaps a little truthfulness in the sense of telling the whole truth. What does the *Star* mean by saying that "it is not 'the medical man' but the advertising physiologist who goes in for vivisection," and flaunting these figures before us, when the self-same table from which these figures are derived shows that of the whole 5,984 inoculations, &c., performed (including those under anæsthetics) only 96 were physiological experiments, all the rest being pathological and therapeutical, that is, directly connected with disease and its treatment.

The sight of suffering of every kind goes against the grain with all of us, but we must maintain a due sense of proportion. The restrictions already imposed on scientific work are, in our opinion, sufficient. Look at the position in which we are already landed. If a medical man meets with a case in which he suspects the presence of poisoning, the law prevents him giving a little of that man's vomit to a mouse to see whether it is poisonous or not! Yet anybody can poison as many mice as he likes so long as he does it not for the sake of saving human life but for sheer destructiveness. We have spoken of the testing of anti-toxins, and of the fact that no one without a licence can test them upon an animal before using them upon a child. But in the preparation of these anti-toxins we believe we are right in saying that no licence is required. Why? Because, forsooth, it is a manufacturing process, and not an experiment! We by no means suggest that sin "in good company" is no longer sin. But we do ask for the matter to be considered from a common-sense point of view, and with some recognition of the urgency of human suffering, and we do protest against the one-sidedness of those who, out of the "ocean of cruelty" with which we find ourselves surrounded, would pick out for suppression that one species of cruelty (of course, we admit the cruelty) which is done for a good purpose, and has for its sole object the mitigation of the sufferings of man.

